# Creating teaching files

**DOI:** 10.2349/biij.2.4.e53

**Published:** 2006-10-01

**Authors:** GL Yang, CCT Lim

**Affiliations:** 1 Biomedical Imaging Laboratory, Agency for Science Technology and Research, Singapore; 2 Department of Neuroradiology, National Neuroscience Institute, Singapore

## INTRODUCTION

Teaching files are useful in the education of diagnostic radiologists. In the course of professional training, radiologists must master a large knowledge base of pictures and develop a method to assess any new, unknown case against that body of information. Imaging-based medical specialties have been traditionally under the purview of diagnostic radiology. However, the practice of diagnostic radiology also includes skilful acquisition of images, such as performing angiography and barium studies, procedures such as diagnostic biopsy or therapeutic interventions guided by imaging. Radiologists also participate in clinical consultation ranging from informal “kerbside” chats with referring physicians to formal multidisciplinary team therapy planning conferences. The bulk of the radiologist’s work however, is based upon the interpretation of images. This article will only deal with teaching files and diagnostic interpretation skills, and not address the other (far more interesting but less defined) skills, nor will it discuss imaging research. [1]

Traditionally, teaching radiology has relied heavily on exposure to the wide range of abnormal cases of different diseases that are available during the course of on-job-training rotations through teaching hospitals. Unfortunately, these abnormal cases are usually hidden among an equally large number of normal studies. Face-to-face teaching, using these abnormal images, usually conveys significant information on interpreting the subtle differences between normal and abnormal, normal mimics of disease, and differentiating different diseases. Most of these mimics have important consequences for treatment and prognosis. These “tips and tricks” tutorial-style encounters are usually part of a larger educational curriculum where formal teaching/lectures are structured around the twin pillars of imaging technique and biology. However, as in all other human activity, “face time” is limited, and access to teachers is limited by their other duties in administration and research, and there may be few opportunities to discuss interesting cases that illustrate a useful point. One important means to make up for this deficit is case-based teaching files.

## RADIOLOGY TEACHING FILES

**Figure 1:**
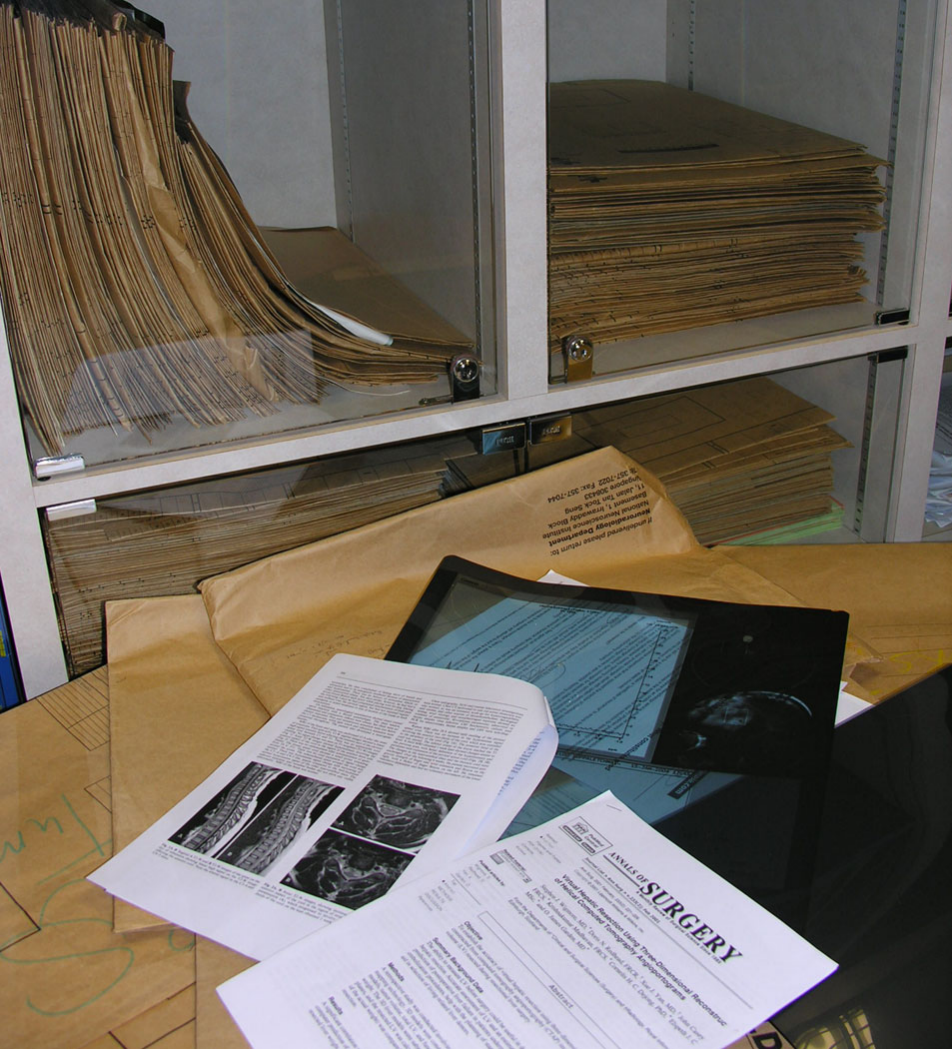
An example of a hardcopy radiology teaching files comprising film images showing the relevant feature, a short write up and (optional) medical literature. In the background is the “library” of teaching files – this can be a challenge to catalogue and maintain.

At its most basic, a teaching file comprises one or more medical images with an important feature that has been created from the radiology studies of a patient, supplemented by relevant clinical data and a short write-up on the pathological condition and/or relevant teaching points (Figure 1). Historically, a simple case-based teaching file comprised the hardcopy film of the image and an accompanying scrap of paper containing at the very least, a correct diagnosis. The amount of information can thus vary from such “bare-bones” files to a full fledged analysis of the subtleties and pitfalls of diagnosis backed by strong evidence in the medical literature, attached for the interested student. The effort required to produce such teaching files also varies, primarily dependent on the technical difficulty of duplicating the clinical image record on to the library film, and the intellectual input demanded of the student. The student is usually one who has benefited from face-to-face teaching, and now carries the responsibility of passing on what he has learnt to future generations.

Often, the problem with teaching files is not so much the creation of the file but the organisation and preservation of many teaching files. Libraries of representative teaching files are highly desirable in radiology teaching programs, but cataloguing and maintenance of these collections for logical and painless retrieval can be problematic. Thus, cases are often filed using classification systems such as the American College of Radiologists (ACR) index of radiological diagnosis; this has several limitations and is already outdated in these latter days of increasingly specific diagnosis using advanced imaging techniques.

## ELECTRONIC TEACHING FILE

**Figure 2:**
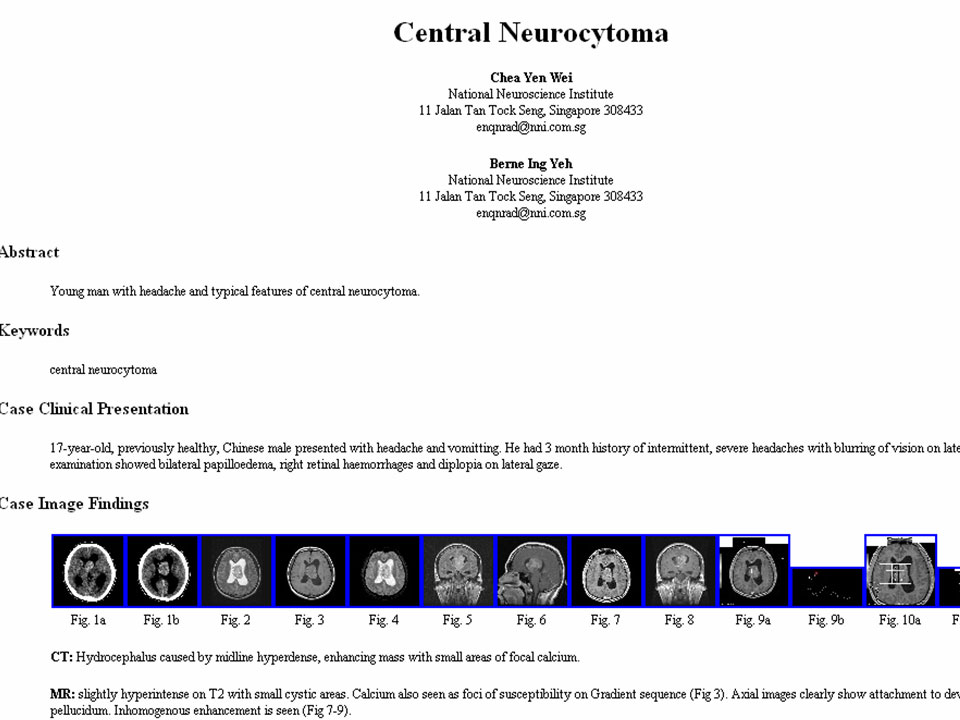
An example of electronic teaching files comprising digital images showing the relevant feature, a short write up and other components of traditional teaching files in an electronic format.

There are many drawbacks to the hardcopy film libraries, such as physical degradation, and single copies that can be misplaced. To overcome these drawbacks and to take advantage of the rich digital information available in picture archive and communications system (PACS), teaching files should move into the electronic realm. Here, the advantages of a computerised structured format and catalogue can be exploited, and many students can access knowledge at their own pace (Figure 2).

**Figure 3:**
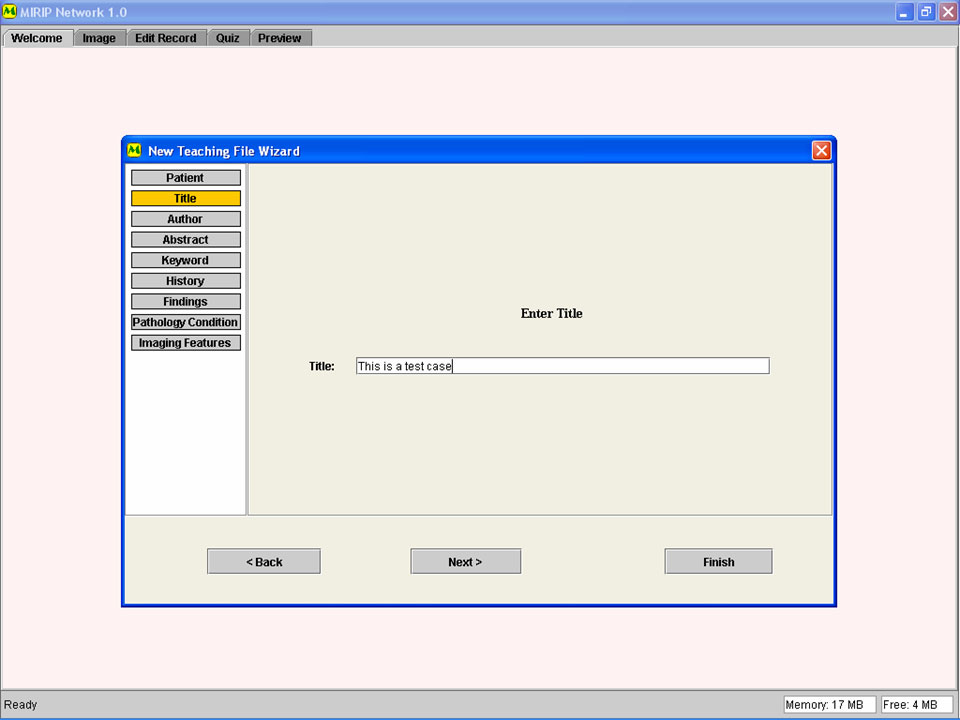
Screen capture of the Singapore National MIRC (medical image repository center) website. This contains a database and web server that allows users to create a structured electronic teaching file in a systematic fashion using the tabs shown on the left (eg. abstract, history, findings, imaging features).

**Figure 4:**
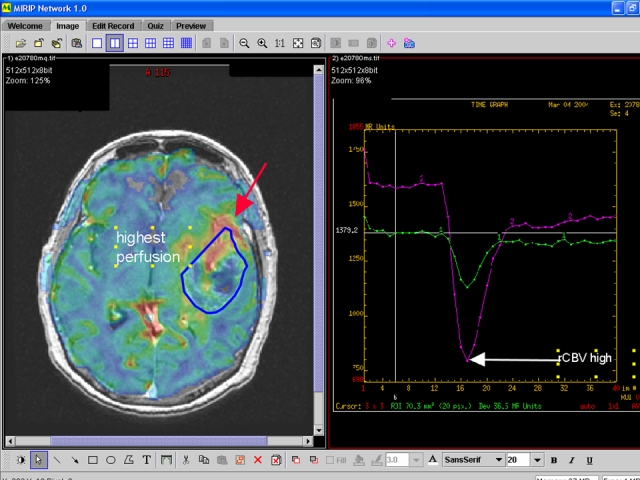
Screen capture of the image creation page for online electronic teaching file. Users may upload an image, and add teaching value by overlaying annotations, arrows and other graphics (found in the bottom toolbar).

**Figure 5:**
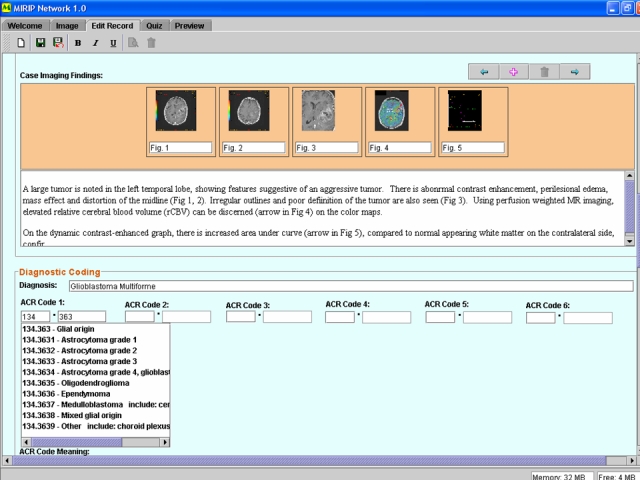
Screen capture of the data entry by form filling and drop-down menus. Free text can be entered to caption the images (“Case Image Findings”) and diagnosis (“Diagnostic Coding”). The appropriate ACR coding index can be selected from a list (arrow).

Our group has previously developed Medical Image Repository Interface with PACS (MIRIP) [2]: a computer server running a database programme (plus an image server and a web server) that enables users in a teaching hospital to create an electronic teaching file from information in PACS. All images are anonymised, and catalogued according to the ACR index. We also reported a suite of authoring tools that functions as a stand-alone collection of electronic teaching files for individuals without hospital PACS [3]. These formats and methods have been expanded to enable participation in the World Wide Web (http://www.mirip.org/nmirc.jsp) [4]. With electronic formatting using XML (extensible markup language) Schema, the process of creating a structured teaching file can be simplified (Figure 3). A teaching file can be broken into its component parts, including the clinical description of the case, the radiology images (captioned by appropriate descriptive text, Figure 4), a short description of the differential diagnosis and discussion of the disease. The information for each part of the structured format may be entered by the user by simply filling in a web-based form (Figure 5). Such a structured method has the advantage of providing a logical and predictable style sheet and at the same time allows some flexibility to encompass different types of images ranging from simple “Aunt Minnies” to complex cases with studies with multiple modalities and differential diagnosis.

## THE WORLD WIDE WEB AND BEYOND: MULTIMEDIA AND DISCUSSIONS

**Figure 6:**
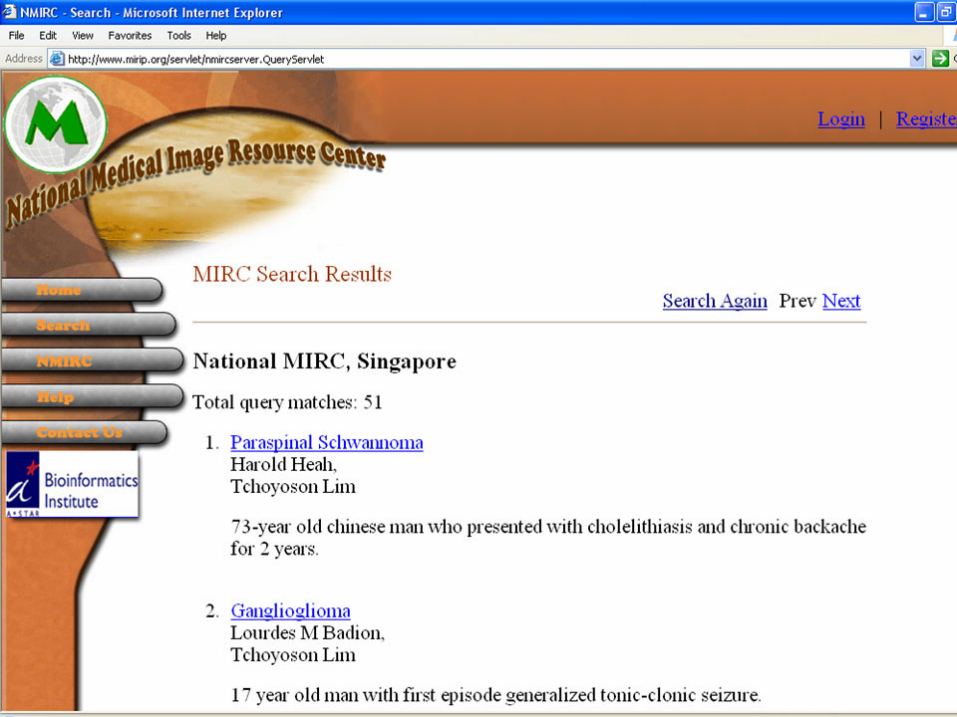
Screen capture of the search feature of a MIRC (medical image repository center) website. This allows users to query the worldwide MIRC websites for keywords (in this case “neoplasm” and see examples of the positive “hits”, each hit being an electronic teaching file.

As far as we are aware, the MIRIP website (http://www.mirip.org) represents the first attempt to establish a collection between electronic teaching files for radiology on the Internet based in Asia. There are many other sites on the Web (http://www.mypacs.net, http://rad.usuhs.mil/medpix, http://www.casimage.com, http://www.auntminnie.com) that offer a “Case of the Day” type electronic teaching files [5]. They also offer some excellent review papers have been published that inform radiologists how they can avail themselves of these resources [6]. Many of these sites offer search engines, and some, such as the Medical Image Repository Center (MIRC) initiative of the Radiological Society of North America (RSNA) (http://mirc.rsna.org) (Figure 6), seek to establish a method to allow the global community of radiologists to share medical images for education and research purposes [7].

Electronic teaching files are desirable as a first step for teaching, and the world of digital image-based education has only just begun to be explored. Multimedia teaching files represent the first step in the dynamic use of audio as well as moving graphics on a radiological image [8]. Furthermore, an image-based discussion forum may be feasible, adding an interactive dimension to teaching that closer simulates the “face-to-face” consultative process that is essential in diagnostic radiology.

## CONCLUSION

Teaching files can be useful for education of radiologists and allied professionals. Face-to-face tutorials for interpretive skills can be supplemented by electronic teaching files, and these can be greatly enhanced by combining PACS and the Internet. We hope that radiologists and allied professionals will participate actively by providing content and expertise for digital teaching files such as online Asian web initiatives (www.mirip.org). These teaching files may form the basis for more interesting and sophisticated aspects of radiological education, such as exploring multimedia education of interpretive and procedural skills.
